# A new genus and species of the family Lepechinellidae Schellenberg, 1926 (Crustacea, Amphipoda) from the Clarion-Clipperton Zone, Central Pacific Ocean

**DOI:** 10.3897/zookeys.1274.140632

**Published:** 2026-03-24

**Authors:** Tammy Horton, Anne-Nina Lörz

**Affiliations:** 1 National Oceanography Centre, Southampton, SO14 3ZH, UK National Oceanography Centre Southampton United Kingdom; 2 Institute of Marine Ecosystem and Fishery Science (IMF), Center for Earth System Research and Sustainability (CEN), University of Hamburg, Große Elbstraße 133, 22767 Hamburg, Germany University of Hamburg Hamburg Germany; 3 Marine Research at Senckenberg am Meer, Südstrand 40, 26382 Wilhelmshaven, Germany Marine Research at Senckenberg am Meer Wilhelmshaven Germany

**Keywords:** Abyss, amphipods, deep sea, *Pseudolepechinella apricity* gen. nov. et sp. nov.

## Abstract

A new monotypic genus within the family Lepechinellidae is described from the deep-sea Clarion-Clipperton Zone in the Pacific Ocean. *Pseudolepechinella***gen. nov**. is distinguished from the other five genera within the family by the rounded/ovoid propodus of the gnathopods, and the unusually short and broadened basis, distally expanded propodus and shortened robust dactyls of the pereopods. The new species is described from two specimens collected more than 180 km apart yet having a 100% COI barcode match. A revised key to the genera within the family is provided.

## Introduction

The family Lepechinellidae Schellenberg, 1926 is a deep-sea family of amphipods (Crustacea, Amphipoda) characterised by elongate, slender pereopods and uropods and a body cuticle covered in long spines and setae (Thurston and Horton 2019; Lörz et al. 2020). Lepechinellids are specialist amphipods with adaptations for the deep-sea environment, including a spinose cuticle with numerous processes on the dorsal midline of the body as well as elongate appendages, both of which are likely adaptations to prevent sinking in soft abyssal sediments (Barnard 1973). Currently, there are five genera in the family Lepechinellidae, three of which (*Lepechinella* Stebbing, 1908; *Lepechinelloides* Thurston, 1980; and *Paralepechinella* Pirlot, 1933) have been reported from the Pacific Ocean (Horton et al. 2024; Peart and Lörz 2026). The remaining genera, *Lepechinellopsis* Ledoyer, 1982 and *Lepesubchela* Johansen & Vader, 2015 are known from the North Atlantic to the Antarctic, the Indian Ocean (Madagascar) and the Northeast Atlantic respectively (Ledoyer 1982; Andres and Brandt 2001; Johansen and Vader 2015). The type genus *Lepechinella* is the most speciose, with 34 species, 13 of which are found in the Pacific Ocean. Only five of these Pacific species have been recorded from depths greater than 3000 m: *Lepechinella sucia* J.L. Barnard, 1961 (3580 m); *Lepechinella uchu* J.L. Barnard, 1973 (3545 m); *Lepechinella ultraabyssalis* Birstein & N. Vinogradova, 1960 (6475–6571 m); *Lepechinella vitrea* Kamenskaya, 1977 (7190 m); *Lepechinella wolffi* Dahl, 1959 (6660–6770 m).

There is a wide morphological variation within the family Lepechinellidae (Barnard 1973; Thurston 1980), and addition of comprehensive molecular information on a wider range of species is desired. Adding molecular information, in addition to the morphological knowledge, will eventually allow a deeper understanding of the relationships both within the lepechinellids and between this family and other amphipod families.

This study adds a new genus and species to the family Lepechinellidae, collected from the Clarion-Clipperton Zone at depths of 4097–4275 m. We provide a diagnosis of the new genus, a description of the new species, and a key to the six genera within the family. We also provide molecular sequence data for the new taxon both as an aid in identification and as a starting point for future evolutionary analysis.

## Methods

The material for the present study was sampled in the central-east Pacific Ocean, specifically in the easternmost sector of the Clarion-Clipperton Zone (CCZ). The material studied was collected using an epibenthic sledge (EBS) and an USNEL spade box corer (Ocean Instruments BX-650; BC) during two expeditions to three different exploration contract areas (henceforth, contract areas) in the CCZ; the UKSR-1 and Ocean Mineral Singapore (OMS) contract areas (ABYSSLINE-2, ABYSSal baseLINE project; Smith et al. 2015), and the NORI-D contract area (Cruise 7A in 2022) following methods in Glover et al. (2015). For details of gear types and sample processing see the relevant cruise reports and Jażdżewska et al. (2025).

The habitus of the holotype specimen is presented as a photograph obtained with a confocal laser scanning microscope (CLSM). The specimen was stained in Congo red and acid fuchsin, temporarily mounted onto slides with glycerol and examined with a Leica TCS SPV equipped with a Leica DM5000 B upright microscope and three visible-light lasers (DPSS 10 mW 561 nm; HeNe 10 mW 633 nm; Ar 100 mW 458, 476, 488 and 514 nm), combined with LAS AF 2.2.1 software (Leica Application Suite, Advanced Fluorescence). A series of photographic stacks was obtained, collecting overlapping optical sections throughout the whole preparation (Michels and Büntzow 2010; Kamanli et al. 2017).

The specimen was then dissected and mounted on temporary slides using glycerol, and illustrations were made using a Nikon SMZ1500 microscope. All slides were examined using either a Nikon Eclipse Ci, or Zeiss compound microscope equipped with a camera lucida. Pencil drawings were scanned and inked digitally using Adobe Illustrator and a WACOM digitiser tablet (Coleman 2003, 2009). Some setae are omitted from the illustrations for clarity. Slight differences are apparent in the illustrated coxae and CLSM owing to the three-dimensional status of this appendage. Appendages of the left side are dissected and illustrated, unless otherwise stated. Specimens were classed as immature (juvenile) when secondary sexual characters (oostegites or penile papillae) were not apparent.

In the descriptions and figures the following abbreviations were used: **A1, A2** = antenna 1, 2; **G1, G2** = gnathopod 1, 2; **H =** head; **LL** = lower lip; **Md** = mandible; **Mx1, Mx2** = maxilla 1, 2; **Mxp** = maxilliped; **P3–P7** = pereopod 3–7; **U1–U3** = uropod 1–3; **UL** = upper lip; **T** = telson; **l** = left; **r** = right.

Type material is deposited in the Senckenberg Museum (Frankfurt, Germany) (**SMF**) and the Natural History Museum, London (**NHMUK**).

### DNA extraction, amplification, and sequencing

DNA from the holotype specimen was extracted and sequenced as described in Jażdżewska et al. (2025). The paratype specimen from the NORI-D area was processed as follows.

DNA was extracted from a pair of pleopods using QuickExtract^TM^ DNA extraction solution (Lucigen), following manufacturer guidelines, and adapted for a digestion time of 45 minutes. Regions of two mitochondrial [16S rRNA (16S) and cytochrome oxidase subunit I (COI)] and early-stage histone 3 (H3)] genetic markers were amplified with published primer sets (Astrin and Stüben 2008; Corrigan et al. 2014; Lörz et al. 2018). The PCR mix for each reaction contained 10.5 µl of Red Taq DNA Polymerase 1.1X MasterMix (VWR), 0.5 µl of each primer (10 µM), and 1 µl of DNA template. Primers and PCR conditions are detailed in Table 1.

**Table 1.** Primers and PCR programs used for DNA amplification.

**Gene**	**Primer**		**Sequence (5' – 3')**	**PCR program**	**Reference**
COI	LCO1490-JJ	Forward	CHACWAAYCATAAAGATATYGG	1 × (2 min at 94 °C), 5 × (30 s at 94 °C, 90 s at 45 °C, 60 s at 72 °C), 35 × (30 s at 94 °C, 90 s at 51 °C, 60 s at 72 °C), 1 × (5 min at 74 °C)	Astrin and Stüben 2008
	HCO2198-JJ	Reverse	AWACTTCVGGRTGVCCAAARAATCA		Astrin and Stüben 2008
16S	16SFt_amp	Forward	GCRGTATIYTRACYGTGCTAAGG	1 × (2 min at 95 °C), 35 × (30 s at 95 °C, 30 s at 50 °C, 45 s at 72 °C), 1 × (5 min at 72 °C)	Lörz et al. 2018
	16SRt_amp	Reverse	CTGGCTTAAACCGRTYTGAACTC		Lörz et al. 2018
H3	HisH3f	Forward	AAATAGCYCGTACYAAGCAGAC	1 × (2 min at 95 °C), 35 × (40 s at 94 °C, 40 s at 45 °C, 40 s at 72 °C), 1 × (10 min at 72 °C)	Corrigan et al. 2014
	HisH3r	Reverse	ATTGAATRTCYTTGGGCATGAT		Corrigan et al. 2014

The primers used for sequencing were the same as those for amplifications. PCR products were purified using a Millipore Multiscreen 96-well PCR Purification System and sequenced using an ABI 3730XL DNA Analyzer (Applied Biosystems) at The Natural History Museum Sequencing Facilities. For each gene fragment contigs were assembled by aligning both forward and reverse sequences, chromatograms were visually inspected, and ambiguous base calls were corrected manually, using Geneious 7.0.6 (Kearse et al. 2012).

Voucher information, taxonomic classifications and sequences are deposited in the data set “DS-AMPHICCZ” in the Barcode of Life Data System (BOLD) (https://doi.org/10.5883/DS-AMPHICCZ) (http://www.boldsystems.org) (Ratnasingham and Hebert 2007).

## Results

### Systematics

**Order Amphipoda Latreille, 1816**

**Suborder Amphilochidea Boeck, 1871**

**Superfamily Dexaminoidea Leach, 1814**

#### Family Lepechinellidae

Taxon classificationAnimaliaAmphipodaLepechinellidae

Schellenberg, 1926

A7F9E147-28AE-5E7F-BE2C-AF9C48B9B30E

Dorbanellidae Schellenberg, 1925: 205.
Lepechinellidae Schellenberg, 1926: 344—K.H. Barnard 1932: 186; Dahl 1959: 235; Andres and Brandt 2001: 79; Sittrop and Serejo 2009: 474; Johansen and Vader 2015: 3. Thurston and Horton 2019: 598.
Lepechinellinae Bousfield & Kendall, 1994: 31―Lowry and Myers 2017: 57.

##### Type genus.

*Lepechinella* Stebbing, 1908, by original designation.

##### Diagnosis.

Body processiferous dorsomedially (variable), and/or covered with setae/spines. One to all coxal plates of pereopods 1–4 acutely pointed distally, sometimes bifid. Urosomites 2 and 3 separate to completely fused. Accessory flagellum one- or two-articulate. Mandibular molar and palp present. Lower lip with inner lobes. Maxilla 2: inner plate without oblique row of setae. Pereopods ***slender*, *elongate to ordinary***; pereopods 5–7 similar, bases linear ***to slightly broadened***. Oostegites narrow. Rami of uropods styloid; uropod 3 outer ramus with tiny second article. Telson normally deeply cleft with lobes gaping widely. ***Amended characters in bold***; after Andres and Brandt 2001; Thurston and Horton 2019).

##### Included genera.

*Lepechinella* Stebbing, 1908; *Lepechinelloides* Thurston, 1980; *Lepechinellopsis* Ledoyer, 1982; *Lepesubchela* Johansen & Vader, 2015; *Pseudolepechinella* gen. nov; *Paralepechinella* Pirlot, 1933.

##### Remarks.

The family Lepechinellidae now includes six genera and 44 species, of which 34 belong to *Lepechinella* (Horton et al. 2024). Depth range: 140–8015 m.

#### Genus Pseudolepechinella gen. nov.

Taxon classificationAnimaliaAmphipoda Lepechinellidae

DED03DE0-DDA1-579F-805B-8C8495362CFD

https://zoobank.org/FDE6BD74-700C-4D31-B379-EE8EEF53BBC5

##### Type species.

*Pseudolepechinella apricity* sp. nov. (monotypy).

##### Etymology.

From the Greek, *pseudos*—false, added to the type genus name, *Lepechinella*, in reference the close resemblance of this species to the genus *Lepechinella*.

##### Generic diagnosis.

Rostrum present; cephalic teeth present; urosomites 2 and 3 fused; mandibular molar triturative; mandibular palp three-articulate; gnathopod 1 carpus elongate, widened distally, propodus strongly rounded/ovoid; gnathopod 2 carpus elongate and slender (as long as basis), propodus short, < 0.25 × carpus, ovoid; Pereopods 6 and 7 simple; pereopods 5–7 bases short, broad, length ~2 × width; pereopods 3–7 propodus widened distally, dactyls short (≤0.5 × propodus), robust; uropods 1–3 outer rami not reduced, longer than 1/3 of inner ramus.

##### Species.

*Pseudolepechinella apricity* sp. nov.

##### Remarks.

The new genus is similar to the type genus *Lepechinella* and would be identified as such if using the most recent key to the Lepechinellidae in Johansen and Vader (2015). While characters of the gnathopods and pereopods are not indicated in previous diagnoses (or keys), these characters will need to be included in subsequent descriptions as increasing morphological variation within the family is being encountered. *Pseudolepechinella* gen. nov. can be distinguished from *Lepechinella* and all other genera within the family by the unusual shape of the propodus of gnathopods 1 and 2 (rounded/ovoid); the extremely elongate, slender carpus of gnathopod 2 (as long as basis and 4 × propodus), the short and more robust form of the pereopod 5–7 bases (length ~2 × width); and the distally expanded propodus with short, robust dactyls on pereopods 3–7. These characters are sufficient to differentiate the new species from all currently described lepechinellid genera and therefore the erection of a new genus for the species is warranted. There are no taxa that possess this combination of unusual gnathopod and pereopod characters within the Lepechinellidae. The majority of lepechinellids are characterised by slender, elongate pereopods, with elongate slender dactyls (length usually ≥ 0.5 × propodus, often subequal to or longer than the propodus), adaptations which are characteristic of amphipods living associated with soft abyssal sediments of the deep sea (J.L. Barnard 1973). The only exception within the family until now is that of the genus *Lepesubchela*, which has subchelate pereopods 6 and 7, suggestive of a clinging life strategy. In the genus *Lepechinella*, there are four species that have some similarities with *Pseudolepechinella* gen. nov. in the form of the shorter more robust pereopod bases: *Lepechinella cachi* J.L. Barnard, 1973; *L. huacho* J.L. Barnard, 1973; *L. occlo* J.L. Barnard; 1973; and *L. pangola* J.L. Barnard, 1962. Of these species, *Lepechinella*occlo and *Lepechinella pangola* are most like *Pseudolepechinella* gen. nov. *Lepechinella occlo* is found in the Pacific, but at much shallower depths of 721 m off New Zealand, while *L. pangola* is found at abyssal depths of 4893 m in the Cape Basin, Atlantic Ocean. However, neither species possesses gnathopods similar to *Pseudolepechinella* gen. nov. and neither the basis broadening of pereopods 5–7 nor the distal expansion of the propodus of pereopod 3–7 are as strong as in the new genus.

### Key to the genera of Lepechinellidae

**Table:** 

1	Mandible palp 1-articulate	***Lepechinelloides* Thurston, 1980**
–	Mandible palp 3-articulate	**2**
2	Pereopods 6 and 7 subchelate	***Lepesubchela* Johansen & Vader, 2015**
–	Pereopods 6 and 7 simple	**3**
3	Mandible palp with article 3 extremely long, much longer than article 2	***Paralepechinella* Pirlot, 1933**
–	Mandible palp with article 3 normal, shorter than article 2	**4**
4	Uropods 1–3 with outer ramus strongly reduced, shorter than 1/3 of inner ramus	***Lepechinellopsis* Ledoyer, 1982**
–	Uropods 1–3 with outer ramus not reduced, longer than 1/3 of inner ramus	**5**
5	Pereopod 5–7 basis slender, elongate, length > 3 × width; gnathopods 1 & 2 propodus subrectangular, gnathopod 2 carpus ordinary, < 3 × propodus	***Lepechinella* Stebbing, 1908**
–	Pereopod 5–7 basis robust, short, length ~2 × width; gnathopods 1 and 2 propodus subovoid, gnathopod 2 carpus elongate, 4 × propodus	***Pseudolepechinella* gen. nov**.

#### Pseudolepechinella apricity sp. nov.

Taxon classificationAnimaliaAmphipodaLepechinellidae

1A3598B1-6249-5354-AB2D-7452FCB9CA56

https://zoobank.org/8B2B12A7-9482-4002-B8CC-38D1B6C82B84

**Figure 1.**
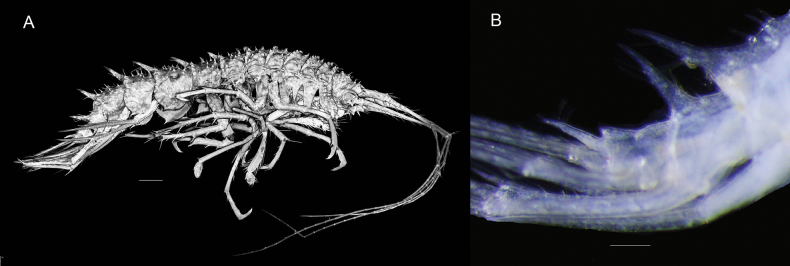
**A***Pseudolepechinella apricity* sp. nov.; holotype SMF 63355, immature, 6.8 mm. CLSM**B***Pseudolepechinella apricity* sp. nov.; paratype NHMUK 2025.26, immature, 7.2 mm. Scale bars: 0.5 mm (**A**); 0.2 mm (Urosome).

**Figure 2.**
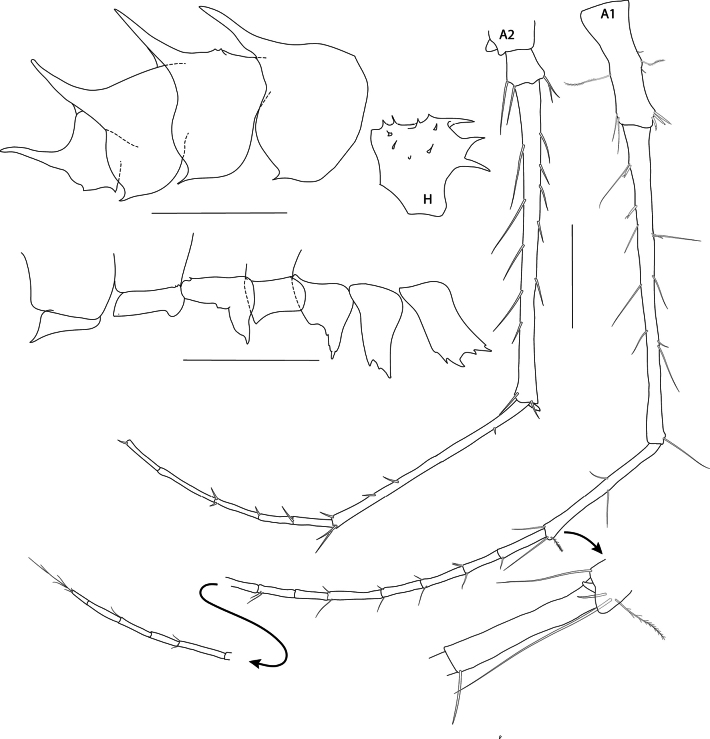
*Pseudolepechinella apricity* sp. nov.; holotype SMF 63355, immature, 6.8 mm. Head, coxae, pleonites, antennae. Scale bars: 1 mm (H, pleonites, coxae); 0.5 mm (Antennae 1–2).

**Figure 3.**
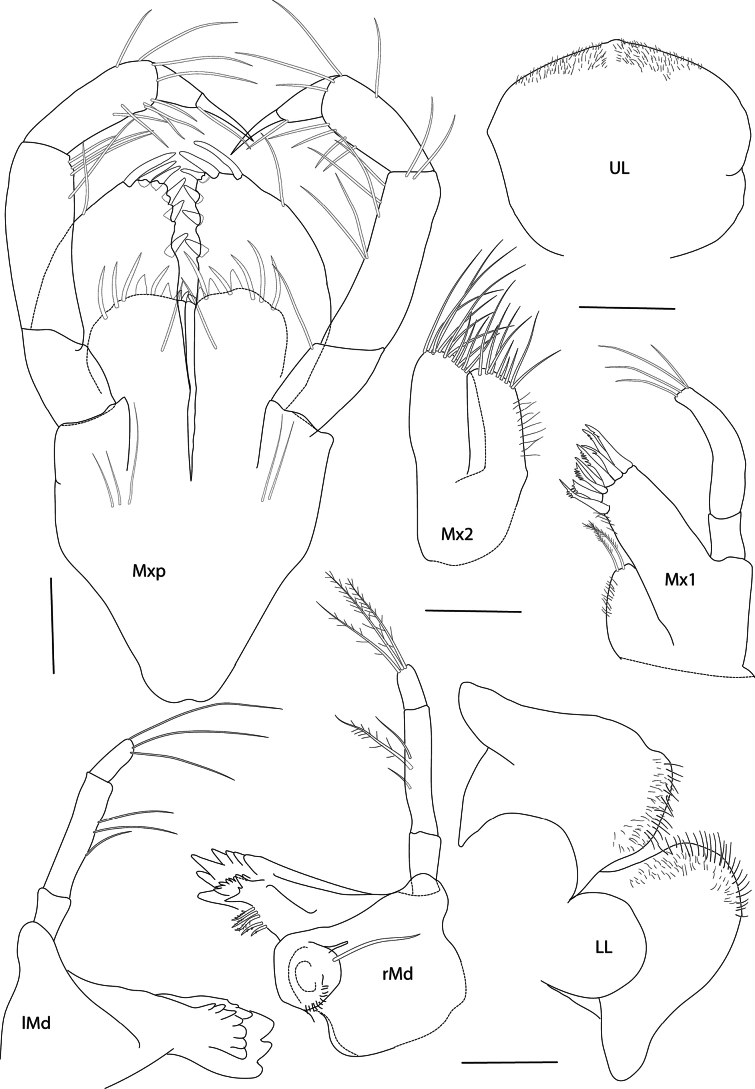
*Pseudolepechinella apricity* sp. nov.; holotype SMF 63355, immature, 6.8 mm. Maxilliped, maxillae 1–2, mandible, lower lip. Scale bars: 0.1 mm.

**Figure 4.**
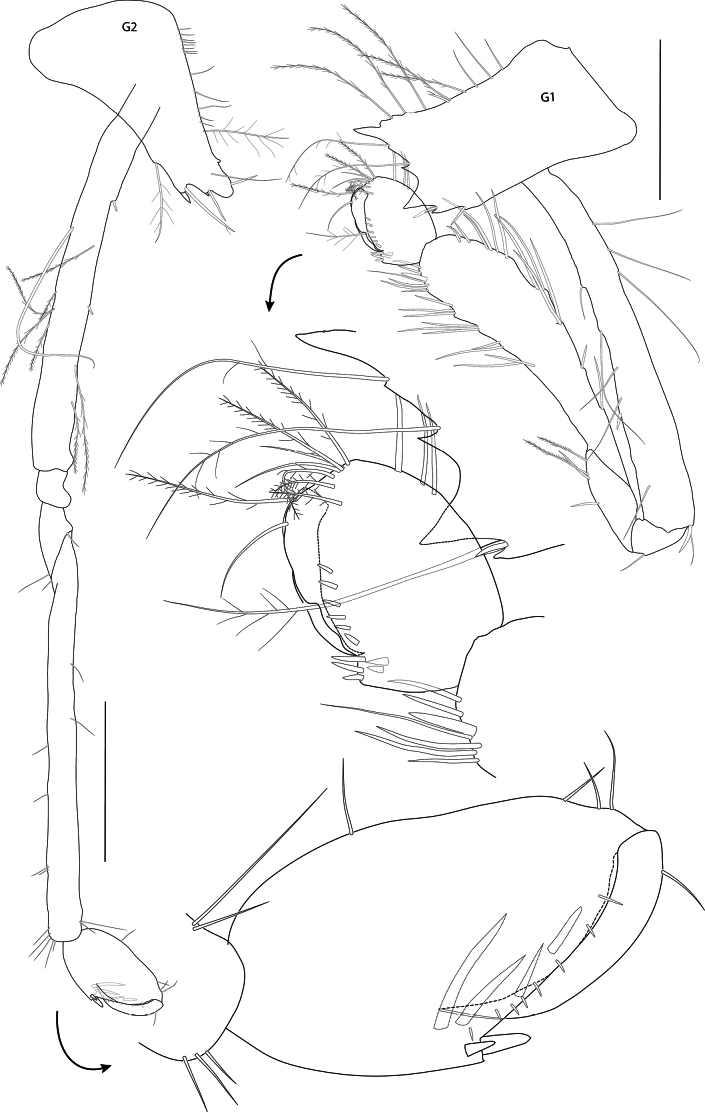
*Pseudolepechinella apricity* sp. nov.; holotype SMF 63355, immature, 6.8 mm. Gnathopods 1–2. Scale bars: 0.5 mm.

**Figure 5.**
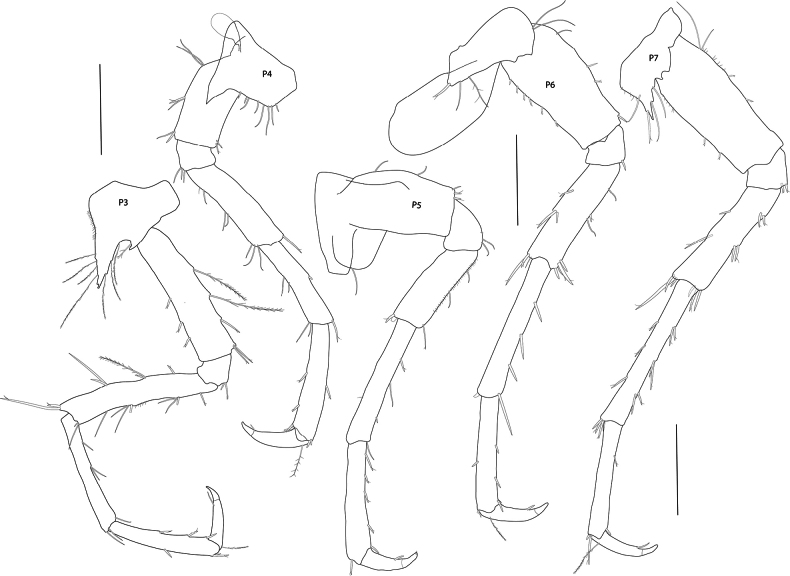
*Pseudolepechinella apricity* sp. nov.; holotype SMF 63355, immature, 6.8 mm. Pereopods 3–7. Scale bars: 0.5 mm.

**Figure 6.**
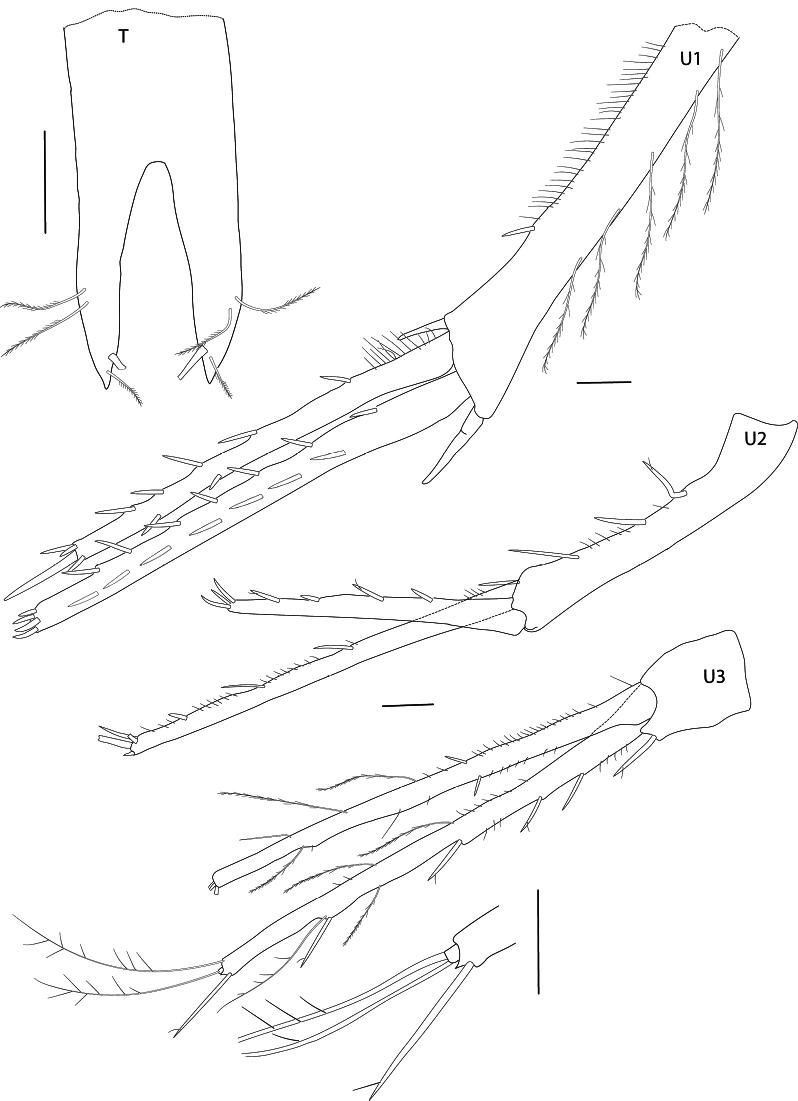
*Pseudolepechinella apricity* sp. nov.; holotype SMF 63355, immature, 6.8 mm. Uropods 1–3, Telson. Scale bars: 0.1 mm (U1–U3, T).

Figs 1, 2, 3, 4, 5, 6

##### Type material.

***Holotype***: Pacific Ocean • immature, 6.8 mm; Clarion-Clipperton Zone; 12.038°N, 117.237°W, 4097 m; 14 March 2015; Ocean Mineral Singapore contract area, RV *Thompson*, ABYSSLINE-2 Cruise, Station AB2-EB11; SMF 63355; COI (PQ734583). ***Paratypes***: Pacific Ocean • immature, 7.2 mm; Clarion-Clipperton Zone; 10.34927°N, 117.169°W, 4275 m; 09 September 2022; NORI-D contract area, MV *Island Pride*, Cruise 7A, Station ES353, Box Core BC_448; Specimen: 9383_TH_AMP_1; NHMUK 2025.26; COI (PV077663); 16S (PV077972), H3 (PV078012) • immature, 8.3 mm; Clarion-Clipperton Zone; 12.339°N, 116.669°W, 4158 m; 09 March 2020; UKSR-1 contract area, MV *Pacific Constructor*, Resource Cruise 01 (RC01), station AOI-3, Box Core BC_28; Specimen: 4082_TH_AMP_1; NHMUK 2025.27.

##### Type locality.

Clarion-Clipperton Zone, 12.038°N, 117.237°W, 4097 m.

##### Etymology.

The species name *apricity* is from the Latin *apricitas*, meaning the feeling of the warmth of the sun in winter, and is used as a noun in apposition. The name is chosen not only to honour the warmth of friendship and laughter, enjoyed in the winter sunshine in Łódź, Poland during the ISA SSKI Amphipod Workshop, but also the bringing to light and warmth from the cold abyssal depths of this new and interesting genus and species of lepechinellid.

##### Diagnosis.

As for the genus.

##### Description.

Based on holotype, immature, SMF 63355, 6.8 mm.

***Body***: (Figs 1, 2): heavily granulated and setose. ***Pereon*** (Fig. 1): pereonites 1–7 with two transverse rows of denticles/small teeth (<20% of height of pereonite), spanning entire anterior and posterior margins of each pereonite, pereonites 6 and 7 with one long posteriorly directed tooth near posterior margin. ***Pleon*** (Figs 1, 2): each segment with long posteriorly directed tooth at posterior margin and several dorsal and dorsolateral denticles/small teeth anterior to main tooth, teeth 50% of height of epimeral plate. ***Epimera*** (Figs 1, 2): separated by a sinus from strongly convex posterior margin with small tooth on disto-ventral angle; tooth and sinus, largest on epimeron 2; each epimeron with lateral row of setae (not illustrated). ***Urosome*** (Figs 1, 2): urosomite 1 produced into strong, posteriorly directed tooth at posterior margin; urosomites 2 and 3 partially fused, with suture, urosomite 2 smooth dorsally, urosomite 3 with low rounded tooth.

***Head***: (Figs 1, 2): granulated and setose; rostrum slightly curved, 20% of length of head; two cephalic projections, curved, acute, as long as rostrum. ***Antenna 1*** (Figs 1, 2): as long as body; first article of peduncle a third of the length of second; third article slightly longer than half the length of second; flagellum 1.5 × peduncle, 25-articulate; accessory flagellum short, one-articulate (likely damaged; in paratype NHMUK 2025.26 the accessory flagellum is as long as flagellum article 4, and two-articulate, second article short). ***Antenna 2*** (Figs 1, 2): subequal to antenna 1; article 5 80% length of article 4; flagellum as long as peduncle articles 1–4; damaged.

***Mouthparts***: (Fig. 3): ***upper lip*** asymmetrically rounded, setulose apically. ***Mandible*** (Fig. 3): incisor process dentate; left ***lacina mobilis*** bluntly dentate, laminar; right acutely dentate, teeth in two rows; molar ridged, triturative; palp as long as body of mandible, first and third articles similar in length, each a third as long as second article each; long, setose setae at apical tip of mandible palp. ***Lower lip*** (Fig. 3): inner lobes well developed, broad, outer lobes setose at apical margins. ***Maxilla 1*** (Fig. 3): inner plate small, slender with two setulose setae apically; outer plate with eight dentate spine teeth distally; palp with four long setae apically. ***Maxilla 2*** (Fig. 3): inner plate slightly shorter and more slender than outer, both plates setose apically. ***Maxilliped*** (Fig. 3): inner plate broad, with four stout and five slender setae apically; outer plate ovo-rectangular, three stout spines medially and three longer spines distally; palp second article slender, as long as third and fourth articles combined, unguis of dactyl as long as basal part.

***Pereon*: *gnathopod 1*** (Fig. 4): subchelate, coxa strongly bifid, a small additional projection posterior of each of the drawn out ventral margins, resulting in four ventral tips of coxa 1, anterior and posterior margins of coxa 1 parallel; basis slender, elongate, margins subparallel, length equal to carpus and merus lengths combined; carpus long, widened distally, weakly setose on both margins; propodus rounded/ovoid, strongly setose, palmar margin marked by three robust spines. ***Gnathopod 2*** (Fig. 4): subchelate, coxa tapering, distally slender; basis and carpus both slender, very elongate, similar in length; merus drawn out posteriorly; propodus short, < 0.25 × carpus, suboval, palm gently convex, palmar margin marked by two stout spines. ***Pereopod 3*** (Figs 1, 2, 5): coxa bifid, strongly produced antero-distally, long slender setulose setae on ventral and posteroventral margins; basis and merus subequal, carpus slightly shorter; propodus slightly shorter than carpus, expanded distally; dactylus short, robust, curved at tip, 0.52 × propodus, with a spine at unguis separation. ***Pereopod 4*** (Figs 1, 2, 5): coxa posterior lobe strongly produced, acute; basis short and broad, length 2.1 × width; distal articles as for pereopod 3, but pereopod more widened distally and dactylus more robust. ***Pereopod 5*** (Figs 1, 2, 5): coxa produced at antero-ventral corner, projection slightly shorter than that of coxa 3; basis short and broad, length 1.8 × width; merus shorter and wider than carpus, carpus longer than propodus; dactylus 0.45 × propodus. ***Pereopod 6*** (Figs 1, 2, 5): coxa with distinct triangular projection at antero-ventral corner; basis length 2.1 × width; distal articles as for pereopod 5. ***Pereopod 7*** (Figs 1, 2, 5): coxa strongly produced postero-distally, ventral margin with four smaller projections; ventral margin with three long serrated setae; basis length 2.4 × width; distal articles as for pereopod 6, but carpus, propodus and dactylus slightly longer and more slender.

***Urosome***: (Figs 1, 6): ***uropod 1*** (Figs 1, 6): peduncle as long as inner ramus, strong spines at distal ends of peduncle, long slender setulose setae at outer margin; inner ramus with spines on both margins; outer ramus slightly longer than inner ramus, spines on both margins. ***Uropod 2*** (Figs 1, 6): peduncle as long as outer ramus; outer ramus shorter, 0.71 × inner ramus; both rami with spines on margins and apices. ***Uropod 3*** (Figs 1, 6): peduncle very short with long spine distally; rami slender, inner ramus slightly shorter than outer, with spines and long setulose setae; outer ramus with minute second article bearing two setae. ***Telson*** (Fig. 6): longer than broad; cleft 60% of length, cleft u-shaped; apices each bearing a long stout spine and setulose setae.

##### Remarks.

*Pseudolepechinella apricity* sp. nov. is unique among lepechinellids in the combination of gnathopod and pereopod characters indicated in the remarks for the genus. While *Pseudolepechinella apricity* sp. nov. is placed within the Lepechinellidae, it is worth noting some of the similarities with other taxa here. Bousfield and Kendall (1994) indicated there were internal subgroupings within the Lepechinellidae that might merit formal recognition, pointing to species which exhibit plesiomorphic (atylinid) character states, such as a lack of mid-dorsal teeth on three or more pereonal segments, coxae 1–4 weakly processiferous below, and the pereopod dactylus less markedly elongate (as is seen in *Pseudolepechinella apricity* sp. nov.) than in other lepechinellid species groups. Despite these plesiomorphies, *Pseudolepechinella* gen. nov. clearly belongs in the Lepechinellidae rather than the Atylidae, owing to the possession of a body cuticle covered in long spines and setae (vs lacking spines and setae), elongate, similar pereopods 5–7 (vs not elongate, dissimilar), styloid, elongate rami of uropod 3 (vs lanceolate, not elongate), and widely gaping telson lobes (vs closely appressed). In the same work, Bousfield and Kendall (1994) also moved the abyssal species *Lepechinella aberrantis* (J.L. Barnard, 1962) to the new genus *Aberratylus* Bousfield & Kendall, 1994, within the Atylidae. There are strong similarities in the form of the pereopods between *Pseudolepechinella* gen nov. and *Aberratylus*, which may warrant further study of that family placement.

##### Distribution.

Abyssal Pacific Ocean, Clarion-Clipperton Zone, 4097–4275 m.

##### Molecular data.

Sequence data for the holotype of *Pseudolepechinella apricity* gen. nov. and sp. nov. is deposited in GenBank under accession number PQ734583 (COI). Sequences of the paratype are deposited in GenBank with the following accession numbers: PV077663 (COI), PV077972 (16S), and PV078012 (H3). The species has also received a Barcode Index Number from Barcode of Life Data Systems: BOLD:AEB1213 (https://doi.org/10.5883/BOLD:AEB1213).

## Supplementary Material

XML Treatment for Lepechinellidae

XML Treatment for Pseudolepechinella

XML Treatment for Pseudolepechinella apricity

## Additional information

### Author ORCIDs

Tammy Horton https://orcid.org/0000-0003-4250-1068

Anne-Nina Lörz https://orcid.org/0000-0001-9942-349X

### Data availability

All of the data that support the findings of this study are available in the main text.
